# New alleles of the lin-22/Hairy bHLH transcription factor

**DOI:** 10.17912/micropub.biology.000111

**Published:** 2019-04-19

**Authors:** Maria Doitsidou, Oliver Hobert

**Affiliations:** 1 Centre for Discovery Brain Sciences, University of Edinburgh, Edinburgh, UK; 2 Department of Biochemistry and Molecular Biophysics, Department of Biological Sciences, Howard Hughes Medical Institute, Columbia University, New York, USA

**Figure 1:**
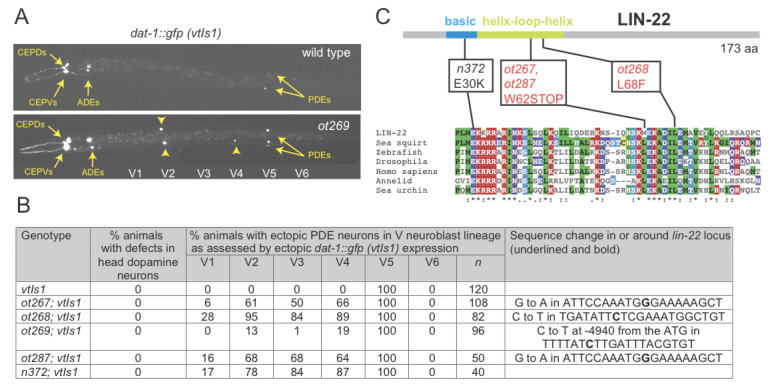
**Alleles of *lin-22.*** (A) *lin-22* mutant alleles display an ectopic expression of *dat-1::gfp (vtIs1;* Nass et al., 2002)*.* One representative example is shown. (B) Quantification of *lin-22* mutant defects and sequence changes. (C) Sequence change in protein coding sequences. Sequences of Hairy homologs from different animal phyla are shown.

## Description

We screened for mutants that affect expression of dopaminergic neuron identity, using a transcriptional reporter for expression of the dopamine transporter *dat-1*. We previously published and characterized a number of mutants that affect *dat-1* expression in different neuron types (Doitsidou et al., 2008). Four alleles that we did not publish in our original screening paper are described here. While wild-type animals only display a single *dat-1::gfp(+)* neuron pair in the midbody region, the PDE neuron pair from the postdeirid lineage, all 4 mutant alleles display ectopic *dat-1::gfp* expression along the anterior/posterior axis of the animal (Fig.1A,B). Postdeirid lineage duplication defects were previously described in animals lacking the bHLH transcription factor *lin-22/Hairy* (Wrischnik and Kenyon, 1997). We find that the canonical *lin-22* allele, *n372*, indeed displays *dat-1::gfp* expression defects similar to those observed in our mutants (Fig.1B). We sequenced the *lin-22* locus in all of our four, independently isolated alleles. Two of them are premature stop codons, one is a missense mutation affecting a conserved leucine residue and all display a similar penetrance of defects (Fig.1B,C). The fourth and weakest allele, *ot269*, displayed no sequence alteration in the *lin-22* coding sequence or in exon/intron boundaries. *ot269* failed to complement *ot267, ot268, ot287* and the canonical *lin-22* allele *n372.* Furthermore, the *ot269* phenotype was rescued by injection of the fosmid WRM0627dG07, which contains *lin-22* and one additional complete gene. We found that *ot269* harbors a single nucleotide change in the upstream intergenic region of *lin-22*, almost 5kb away from the start of the gene (sequence change shown in Fig.1B). Subsequent work has shown that this mutation affects a binding site for a GATA transcription factor (Katsanos et al. 2017).

## Reagents

OH4265 *lin-22(ot267);vtIs1*

OH4270 *lin-22(ot268);vtIs1*

OH4271 *lin-22(ot269);vtIs1*

OH4320 *lin-22(ot287);vtIs1*

Strains are available at the CGC.
